# Patients with an acetabular fracture treated with acute total hip arthroplasty and additional fixation: a cohort study with functional and radiological follow-ups

**DOI:** 10.2340/17453674.2026.45870

**Published:** 2026-05-29

**Authors:** David CHANG, Seppo K KOSKINEN, Anders ENOCSON

**Affiliations:** 1 Department of Molecular Medicine and Surgery, Karolinska Institute, Department of Trauma and Orthopaedics, Karolinska University Hospital, Stockholm; 2 Department of Clinical Science Intervention and Technology, Karolinska Institute, Department of Radiology, Karolinska University Hospital, Stockholm, Sweden

## Abstract

**Background and purpose:**

Elderly patients with acetabular fractures are associated with complex fracture patterns and poor bone quality. Internal fixation alone in these patients has been associated with poor outcome. Internal fixation combined with acute total hip arthroplasty (THA) has been introduced as a surgical option aimed to allow early recovery of hip function and mobility. However, functional and radiological data are still limited. The aim of our study was to elucidate radiographic and functional outcomes, and complications in elderly patients with an acetabular fracture treated with acute THA and additional fixation (reinforcement ring and/or plate fixation).

**Methods:**

Patients ≥ 60 years with an acetabular fracture treated with acute THA and additional acetabular fixation were followed up at least 1 year postoperatively. Medical records were reviewed, and functional examinations and radiographic assessments were performed.

**Results:**

84 patients were identified, of whom 32 completed all follow-ups. The median (IQR, range) age was 77 (35, 60–95) years and 7 of 32 were females. Dome impaction was the main indication for THA in 30 and the median follow-up time was 3.1 years. 2 patients had a postoperative hip dislocation. 3 patients had a deep infection and 7 sustained at least 1 non-surgical complication. The median Harris Hip Score was 87 (33, 33–100) and the median EQ-5D-3L index score was 0.7 (0.5, 0.0–1.0). No radiological signs of loosening of the acetabular reinforcement ring or the cup were observed. The 1-year mortality for the whole eligible cohort was 13% (n = 9/72).

**Conclusion:**

Internal fixation with acute THA in elderly acetabular-fracture patients resulted in good functional outcomes with no signs of acetabular implant loosening, but a high complication rate.

Displaced acetabular fractures in the elderly are associated with severe morbidity and increased risk of mortality [1]. In addition, these injuries have been difficult to treat with open reduction and internal fixation (ORIF) alone due to weak bone, which may lead to implant failure and subsequent need for secondary conversion to total hip arthroplasty (THA) [2,3]. Predictors for poor prognosis after ORIF include substantial impaction or comminution of the weightbearing surface/dome and/or posterior wall fracture patterns [4]. Furthermore, delayed THA has been associated with more technical difficulties as patients tend to develop significant scarring in combination with altered anatomy [5]. To circumvent the issue of insufficient fixation of the acetabular fracture and late posttraumatic complications, the method of combining internal fixation with an acute primary THA has been proposed [5], sometimes with the use of an acetabular reinforcement ring [6,7]. The major benefit is early mobilization with full weightbearing and the potential to rehabilitate the frail elderly more promptly after surgery, thereby avoiding complications associated with inactivity [8]. However, acetabular fracture surgery with THA may be complex, and it is associated with complications [9]. In addition, the current literature on functional and radiological outcomes in these patients mostly consists of limited series with relatively short follow-up times [10]. Therefore, the aim of our study was to elucidate radiographic and functional outcomes in elderly patients with an acetabular fracture treated with acute THA and additional fixation (reinforcement ring and/or ORIF).

## Methods

### Study design

This was a descriptive, prospective cohort study with clinical and radiological follow-ups. The study was reported according to the STROBE guidelines.

### Patients

All elderly patients with an acetabular fracture treated with a combination of acetabular fracture fixation and THA between 2017 and 2023 at the Karolinska University Hospital were identified in the clinical database. Some results, but no functional or radiological data, for the original cohort have been published previously [11]. All the patients were operated on by senior consultant orthopedic surgeons with many years of experience in acetabular/pelvic fracture surgery as well as THA. Inclusion criteria for the study were age from 60 years at the time of surgery, surgery within 21 days after the injury, and a combination of primary THA and additional acetabular fracture fixation (plate fixation and/or reinforcement ring). Exclusion criteria were pathological acetabular fracture, periprosthetic acetabular fracture, inability to attend a follow-up due to geographic inconvenience, cognitive impairment, drug abuse, or any severe medical impairments that hindered attendance. All eligible patients were offered a follow-up, at a minimum of 1 year postoperatively. The follow-up consisted of clinical examination as well as assessment of functional and radiological outcomes. In addition, patient records, including previous radiographs, were analyzed. Collected parameters consisted of patient-related characteristics, injury-related data, reoperations, and complications. An infection was defined as a combination of clinical findings and positive cultures from the operation site. All fractures were analyzed with a preoperative computed tomography (CT) scan and classified according to Judet et al. [12] by 2 of the authors (DC and AE).

### Functional assessments

The patients’ hip function was assessed using the Harris Hip Score and their health-related quality of life by the EQ-5D-3L. Harris Hip Score is a functional scoring system, which includes a patient-derived report of dysfunction in daily life as well as clinical examination of range of motion. It generates a score up to 100 points where 90–100 points corresponds to excellent function, 80–89 good, 70–79 fair, and < 70 poor ]13]. EQ-5D-3L assesses 5 different areas/dimensions pertaining to health-related quality of life such as mobility, self-care, usual activity, pain or discomfort, anxiety or depression. These dimensions can be used to generate the EQ-5D-3L index score, a numerical value from 0 (worst possible health) to 1 (best possible health) [14].

### Radiographic assessments

Postoperative anteroposterior (AP) pelvic, AP and a groin-lateral radiographs of the operated hip were analyzed. The variables analyzed included heterotopic ossification (HO) around the hip [15] and the presence of radiolucent lines between the bone and cement in both acetabular and femoral components of the prosthesis. The cup/reinforcement ring was divided into 3 zones and the femoral stem into 7 zones according to DeLee a Charnley [16] and Gruen [17]. The radiographs were evaluated chronologically to assess any progressive changes in the width and/or length of radiolucent zones and a dichotomous scale was used, i.e., radiolucent zone was not present vs radiolucent zone was present on radiographs obtained at the time of the last clinical follow-up. A senior radiologist with more than 25 years of experience in musculoskeletal radiology (SK) analyzed the radiographs using PACS workstation (Sectra PACS IDS7, v.23.1, Linköping, Sweden). The imaging and clinical data were neither blinded nor anonymized.

### Statistics

The numerical data were presented as median (IQR; range) and the categorical data as frequencies and/or percentages. The Shapiro–Wilk test for normality of data was used. The analyses were conducted using IBM SPSS Statistics, version 29.0 (IBM Corp, Armonk, NY, USA).

### Ethics, data sharing plan, funding, use of AI, and disclosures

This study was approved by the Swedish Ethical Review Authority, Ref. Number 2023-05170-01. Written informed consent was collected from all study patients. The datasets used during and/or analyzed during the current study are available from the corresponding author on reasonable request. The study was funded by Promobilia (grant number A23117). No use of AI was made during the writing process of this study. No conflicting interests were declared. Complete disclosure of interest forms according to ICMJE are available on the article page, doi: 10.2340/17453674.2026.45870

## Results

### Patient characteristics

**Figure 1:**
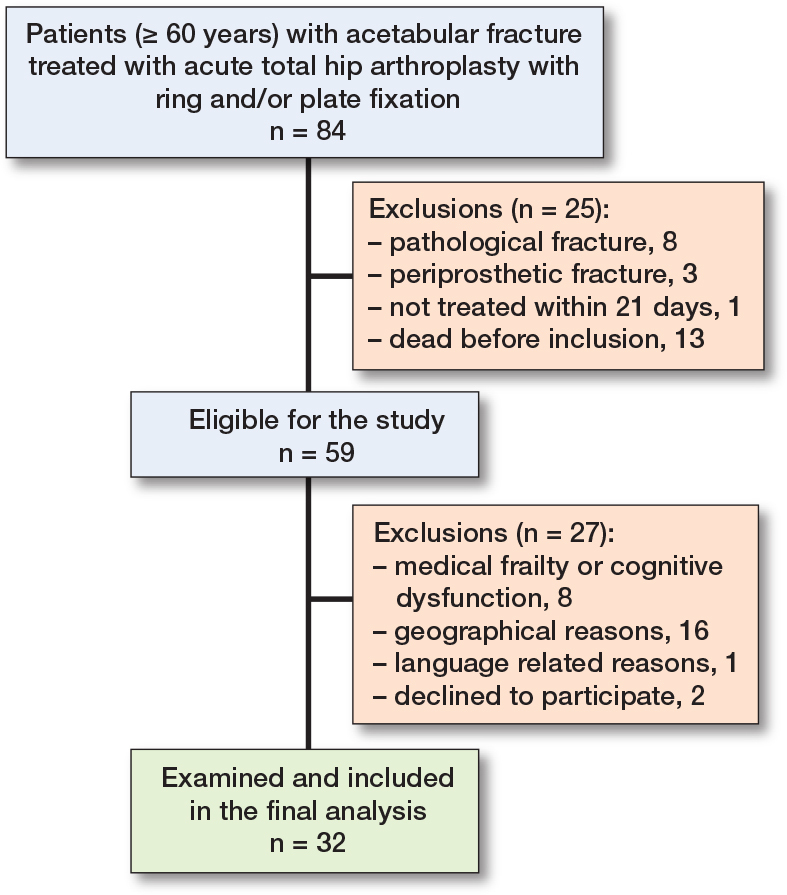
Flowchart of the patient selection process.

Initially, 84 elderly patients operated on with a combination of acetabular fracture fixation and an acute THA were identified. 12 patients were excluded due to not fulfilling the inclusion criteria (8 patients with a pathological acetabular fracture, 3 patients with a periprosthetic acetabular fracture, and 1 patient who was operated on 22 days after the injury). Another 13 patients were deceased (9 within the first year). Of those, 27 patients were excluded due to medical frailty, cognitive dysfunction, geographical reasons, language difficulties or declined to participate. The final number of patients that attended the follow-ups was 32, and data was complete for all outcomes (Figure 1).

**Figure 2:**
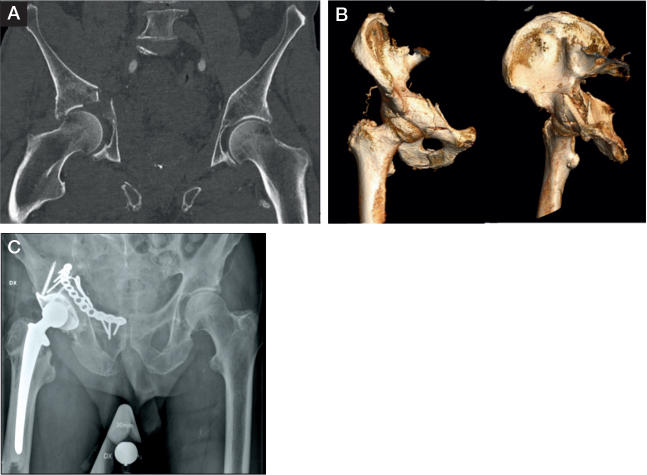
Patient with anterior column + posterior hemitransverse fracture, and medial protrusion of the femoral head. (A) Dome impaction in CT, (B) 3D reconstruction, (C) treated with plate fixation, reinforcement ring, and acute THA.

The median age at the time of surgery was 77 (35, 60–95) years and the cohort consisted of 7 females. The ASA class was ASA II (n = 15) or ASA III (n = 17). The majority of the fractures were the result of a low-energy trauma (n = 25) due to a simple fall (n = 24). Some patients sustained associated injuries, including chest injury (n = 2), major upper limb injury (n = 2), head injury (n = 1), and/or major lower limb injury (n = 1). The most common acetabular fracture patterns were anterior column + posterior hemitransverse (n = 14), associated both column (n = 8), and anterior column (n = 7) (Figure 2). The majority of the patients had major dome impaction (n = 30) as the surgical indication for THA rather than fracture fixation only. 15 of the patients were operated in within 72 hours from hospital admission, and the median hospital length of stay was 6 days (5; 2–30). The median follow-up time was 3.1 (2.4; 1.0–6.9) years. The 1-year mortality for the whole eligible cohort was 13% (n = 9/72) Additional patient and injury characteristics are presented in Table 1.

**Table 1:** Patient characteristics, injury- and treatment-related variables (N = 32). Values are count (%) unless otherwise specified

Variable	Value
Age, median (IQR, range)	77 (35, 60–95)
Female sex	7
ASA class	
II	15
III	17
Mechanism of injury	
Simple fall	24
Fall from height	4
Bicycle accident	4
Associated injuries	
Head injury	1
Chest injury	2
Major upper limb injury	2
Major lower limb injury	1
Judet–Letournel fracture type	
Anterior column	7
Posterior wall	2
Anterior column + posterior hemitransverse	14
Both columns	8
Unable to classify	1
Indication for THA	
Dome impaction	30
Posterior wall injury	2
Operated on within 72 hours	15
Days of hospital stay median (IQR, range)	6 (5, 2–30)

ASA = American Society of Anesthesiologists Physical Status classification; IQR = interquartile range.

### Treatment

All patients were treated with a cemented THA with a Lubinus SPII stem (Waldemar Link, Hamburg, Germany) and a Lubinus SPII cup (n = 3) or Marathon cup (n = 29) (DePuy Synthes, Richmond, VA, USA). The most common acetabular fracture fixation was a combination of an anterior plate with a reinforcement ring (n = 16) and the most commonly used ring was a Müller ring (n = 29) (Zimmer Biomet, Warsaw, IN, USA) (Figure 2C). Further overview of fracture patterns and their corresponding fixation type is summarized in Table 2. The hip prosthesis approaches used were an anterolateral (Gammer) (n = 26) or a posterior approach (n = 6), all in lateral decubitus position. All surgical procedures were performed at the same time, with a median total operation time (including time for change of patient position in some cases) of 236 (116; 105–390) min. The median perioperative blood loss was 700 (825; 300–1,900) mL. Perioperative intravenous antibiotic prophylaxis was given to all patients, as well as postoperative low-molecular-weight heparin as thromboembolic prophylaxis (normally for 6 weeks, shorter for patients on regular other antithrombotic agents).

**Table 2:** Type of fixation in relation to fracture type. Values are count

Fracture type	Ring	Fixation type			Posterior plate	Total
		Ring + anterior plate	Ring + posterior plate	Ring + double plates		
Anterior column	3	4	0	0	0	7
Posterior wall	0	0	1	0	1	2
Anterior column + posterior hemitransverse	5	9	0	0	0	14
Associated both column	1	2	4	1	0	8
Unable to classify	0	1	0	0	0	1
Total	9	16	5	1	1	32

### Complications

Reoperations occurred in 2 patients due to hip dislocation, of whom 1 was operated on by a posterior approach with a Burch-Schneider ring (Zimmer Biomet, Warsaw, IN, USA) and the other by an anterolateral approach with a Müller ring. Both patients went through 2 closed reductions and subsequently underwent cup revision (1 with a constrained cup and 1 with a new repositioned cup) as the final treatment. Deep infection was detected in 3 patients with 2 of them occurring more than 1 year postoperatively, and 1 within the first week. Subsequently, all of them were treated with long-term antibiotics only as 1 patient rejected surgery and 2 were judged to be too frail for further extensive revision surgery. Moreover, 7 of the patients sustained at least one non-surgical complication which included 4 cases of pulmonary embolism, 1 case of pneumonia, 1 case of urinary tract infection, and 1 case of deep venous thrombosis.

### Radiographic outcomes

Signs of stem loosening according to Gruen zones were observed in 8 patients, with zone 1 as the most common area (n = 6). 3 patients showed multiple zones of radiolucency. In contrast, none of the patients showed signs of acetabular cup or reinforcement ring loosening. Heterotopic ossification was observed in 30 of the patients with most of them being Brooker grade 1 (n = 18), and only 1 patient with grade 4 (Table 3).

**Table 3:** Radiological findings of stem loosening (Gruen zones) and heterotopic ossification (Brooker classification) at follow-up (N = 32)

Variable	Patients, n
Stem loosening	
Zone 1	6
Zone 2	1
Zone 3	1
Zone 4	0
Zone 5	0
Zone 6	1
Zone 7	4
Multiple zones	3
Heterotopic ossification	
Grade 1	18
Grade 2	5
Grade 3	6
Grade 4	1

### Functional outcomes

The median Harris Hip Score for all patients was 87 (33; 33–100). 14 patients were considered as excellent, 6 as good, 3 as fair, and 9 as poor. The median EQ-5D-3L index score for all patients was 0.7 (0.5; 0.0–1.0), where 20 of the patients reported some difficulties in walking, 18 some pain/discomfort, and 16 some anxiety/depression. Among the patients who reported extreme difficulties, self-hygiene (n = 4), and pain (n = 4) were the most commonly affected domains (Table 4).

**Table 4:** Overview of functional outcomes at follow-up (N = 32). Values are count unless otherwise specified

Variable	Value
Harris Hip score, median (IQR; range)	87 (33; 33–100)
Excellent (≥ 90)	14
Good (80–89)	6
Fair (70–79)	3
Poor (< 70)	9
EQ-5D-3L index score, median (IQR; range)	0.7 (0.5; 0.0–1.0)
Mobility	
No difficulty	10
Some difficulties	20
Extreme difficulties	2
Self-care	
No difficulty	21
Some difficulties	7
Extreme difficulties	4
Usual activities	
No difficulty	19
Some difficulties	10
Extreme difficulties	3
Pain/discomfort	
No difficulty	10
Some difficulties	18
Extreme difficulties	4
Anxiety/depression	
No difficulty	16
Some difficulties	16
Extreme difficulties	0

**Figure 3:**
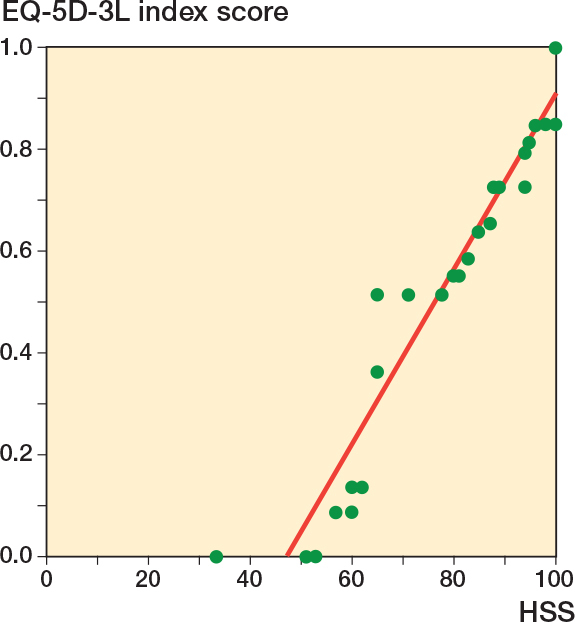
Scatter plot of EQ-5D-3L index score as a function of HHS.

A positive correlation between HHS and EQ-5D-3L index score was found (ρ = 0.8, P = 0.001, n = 32), suggesting that higher HHS was associated with higher EQ-5D-3L index score (Figure 3).

## Discussion

In our study, the functional and radiographic outcomes of elderly acetabular fracture patients treated with internal fixation and acute THA were investigated. The main findings were good function and the absence of acetabular component loosening, but a high complication rate.

The fracture patterns and methods of combining fixation with THA among the patients were slightly heterogeneous. Most of the patients were operated on with a reinforcement ring in combination with or without additional plating. Despite this, none of the patients showed loosening of either the ring or the cup, even among patients followed up to 6.9 years postoperatively, which suggests long-term durability of the acetabular component, as indicated by a previous prospective series with 4 years’ follow-up [7]. Although radiolucency around the femoral stem was observed in 25% of patients, most of them did not show engagement of multiple zones and therefore did not necessarily correlate with clinically significant loosening [18]. HO was observed in 30 patients, with 6 having grade 3 and 1 grade 4. However, no correlation between HO and HHS or EQ-5D-3L index score could be observed, which might be due to insufficient patient numbers or the fact that most patients only had a grade 1 HO.

We found a median HHS of 87, which corresponds to good function, but still 9 of the patients had HHS < 70 (poor function). In a recent study, Kirkeboe et al. reported a mean HHS of 83 at 1 year in 36 patients, but a lower share with poor function (11%) [19], an important difference being that their cohort was younger (mean age 63 vs median 77), with the youngest patient being only 37 years old. In a 2022, systematic review by McCormick et al. the outcomes of different treatments for acetabular fractures in the elderly were compared by pooling 38 studies [20]. An average HHS of 85 was reported among the ORIF+THA group, which was comparable with the results of our study. In general, our numbers align with previously reported results in the literature, but there seems to be a subpopulation of patients that do report poor function [10,20–23]. The reason for this phenomenon is unknown, and future studies should hopefully help clarify this, and thereby make it possible to improve the outcome for all these patients. Furthermore, when compared with patients with a hip fracture treated with THA and similar follow-up time, the mean HHS was similar with a score of 87 at 1 year postoperatively and 89 at 4 years among the hip fracture patients in a 2011 RCT by Hedbeck et al. [24]. In addition, the EQ-5D-3L score for both this study and the study by Hedbeck et al. was similar at 1 and 4 years postoperatively. The correlation between the HHS and EQ-5D-3L in this study indicates a concordance between the values from the patients’ self-reporting and those derived from the clinical examination. As many of the patients reported at least some pain/discomfort and anxiety/depression, it illustrates the importance of additional long-term postoperative rehabilitation and care.

We found that 7 of the 32 patients had a non-surgical complication, of whom 4 patients had a venous thromboembolic event. This is a potentially serious event, and they occurred although prophylaxis was used by all patients for quite a long time (6 weeks) postoperatively. In addition, 5 had a surgically related complication with 2 cases of hip dislocation. In comparison, McCormick et al. reported a complication rate of 25% for ORIF+THA patients, which was still lower than for ORIF alone (38%) and with an OR of 1.87 [20]. The rate of dislocations was comparable to a previous report on ORIF+THA patients where Basset et al. reported a 6% dislocation rate in a cohort of 51 patients [25]. In contrast, Liang et al. reported up to 12% dislocations in a 2023 meta-analysis with a total of 255 patients [26].

The mortality of the entire cohort was surprisingly low (13%, n = 9/72, at 1 year) considering the patient selection (median age 77 years and > 50% ASA class 3). As a comparison, Ljungdahl et al. recently reported 15% 1-year mortality in surgically treated, and 21% in non-surgically treated, geriatric patients with acetabular fractures [27]. Although auspicious, the low mortality among surgically treated patients most likely represents a selection of patients being fit enough for surgery. This is partly supported by the findings in a study by Stetzelberger et al. who found almost twice as high mortality (18% vs 33%) in hip fracture patients (who are almost always operated on) compared with surgically treated geriatric acetabular fracture patients [28]. However, as this study focused on functional and radiographical outcomes, and thus only included patients who were alive, further analysis on this topic is better analyzed in other studies [11].

In the literature, several different implant combinations have been used for this type of surgery [10]. In our series, most of the cases were operated on with an acetabular ring (cage), which was fixed with screws to act as an augmentation and foundation for a cemented cup. However, all the implants used were chosen at the discretion of the surgeon and based on the actual case. Zhang et al. reported in a systematic review with 33 studies that similar acetabular implants (ring/cage) were used in about half of the series [29]. Although the ring displayed good results in terms of fixation and stability in this series, it still adds an additional cost to the operation, and future studies on the optimal implant combination will be of great interest.

### Limitations

This is a single center study, with a low patient-cohort size and many excluded patients. This in turn may skew the data towards examining only the patients that are motivated and healthy enough to attend the follow-ups. The number of dropouts due to medical conditions and/or cognitive dysfunction was considerable. Although the 1-year mortality was relatively low, still 13 patients were deceased and more than 20 patients were excluded due to reasons more or less associated with old age and frailty, which is difficult to avoid in this patient group.

### Conclusion

We showed that internal fixation with acute THA among a selected group of elderly patients with acetabular fractures yields a good functional outcome with no signs of acetabular implant loosening, but a high complication rate.

## Notes

All authors contributed to the study conception and design. Material preparation, data collection, and analysis were performed by DC and AE, and radiographic data interpretation by SK.

Luigi Belcastro is thanked for his valuable contribution with patient coordination. The authors would also like to thank the Promobilia Foundation, for generously funding this study.

Handling co-editors: Marianne Westberg and Robin Christensen

*Acta* thanks Jeppe Barckman and other anonymous reviewers for help with peer review of this manuscript.
